# Odds of Incomplete Colonoscopy in Colorectal Cancer Screening Based on Socioeconomic Status

**DOI:** 10.3390/diagnostics12010171

**Published:** 2022-01-12

**Authors:** Birgitte Skau, Ulrik Deding, Lasse Kaalby, Gunnar Baatrup, Morten Kobaek-Larsen, Issam Al-Najami

**Affiliations:** 1 Department of Surgery, Odense University Hospital, 5000 Odense, Denmark; ulrik.deding@rsyd.dk (U.D.); lkm@rsyd.dk (L.K.); gunnar.baatrup@rsyd.dk (G.B.); Morten.Kobaek.Larsen@rsyd.dk (M.K.-L.); Issam.Al-Najami@rsyd.dk (I.A.-N.); 2 Department of Clinical Research, University of Southern Denmark, 5230 Odense, Denmark

**Keywords:** colorectal cancer, screening, inequality, socioeconomic status, comorbidity, incomplete colonoscopy

## Abstract

The aim of this study is to investigate the association between socioeconomic status (SES) and the risk of having an incomplete colonoscopy (IC) in the Danish Colorectal Cancer (CRC) Screening Program. In this register-based study we included 71,973 participants who underwent colonoscopy after a positive fecal immunochemical test in the Danish CRC Screening Program. The main exposure, SES, was defined by income and education, and the outcome by complete or incomplete colonoscopy. Among the participants, 5428 (7.5%) had an incomplete colonoscopy. The odds ratio (OR) for ICs due to inadequate bowel preparation was 1.67 (95% CI: 1.46; 1.91) for income in the 1 quartile compared to income in the 4th quartile. ORs for income in the 2nd quartile was 1.38 (95% CI: 1.21; 1.56) and 1.17 (95% CI: 1.03; 1.33) for income in the 3rd quartile. For the educational level, an association was seen for high school/vocational education with an OR of 0.87 (95% CI: 0.79; 0.97) compared to higher education. For ICs due to other reasons, the level of income was associated with the risk of having an IC with an OR of 1.19 (95% CI: 1.05; 1.35) in the 1st quartile and an OR of 1.19 (95% CI: 1.06; 1.34) in the 2nd quartile. For the educational level, there were no significant associations. Low income is associated with high risk of having an IC, whereas educational level does not show the same unambiguous association.

## 1. Introduction

Colorectal cancer screening (CRC) has been implemented in numerous countries worldwide and often consists of a fecal immunochemical test (FIT) followed by colonoscopy [1]. In the Danish CRC Screening Program, a colonoscopy is considered incomplete when the caecum is not reached, or when the colonic mucosa is not sufficiently visible in all segments [2]. Often, it is incomplete due to inadequate bowel preparation [3,4], but it can also be caused by pain or anatomical differences, such as difficult angulations. There are multiple known risk factors for incomplete colonoscopies (IC), such as gender [4,5,6,7] and low BMI [4,6,8]. Comorbidity and medication are commonly reported reasons for poor bowel preparation. Franco et al. stated that patients suffering from diabetes, patients having undergone abdominal and especially pelvic surgery, or patients taking certain medications, such as opioids, tricyclic medicine and other antidepressants, have an increased risk of poor bowel preparation [4].

In the case of IC, the participants will have a new investigation, either by colonoscopy under general anesthesia or other modalities, such as CT colonography. Thus, an additional bowel preparation is required, which leads to diagnostic delay, inconvenience, discomfort and risk for adverse events for the patient. Further, additional investigations increase the cost per patient reducing cost-effectiveness. The risk of CRC for patients with an IC is almost three times higher compared to patients who had a complete initial colonoscopy [9].

Social inequality in CRC and screening is evident, and even though CRC incidence is higher among people with higher socioeconomic status (SES), survival is better in this segment [10]. SES is known to affect the overall participation rate, the ability to submit eligible FIT samples and the participation in colonoscopy after positive FIT [11,12,13], but the effect on SES on IC has not been investigated. Therefore, we pursue to investigate the socioeconomic inequalities in the Danish CRC Screening Program further. The aim of this study was to estimate the odds of IC based on SES among FIT positive participants in the Danish CRC Screening Program.

## 2. Materials and Methods

### 2.1. Study Population and Design

The Danish CRC Screening Program was launched in 2014 distributing biennial invitations to all Danish citizens aged 50–74. Participation is optional and free of charge [14]. Together with the CRC screening invitation, citizens receive a stool sample test kit for FIT. In case the FIT is positive (≥100 ng hemoglobin/mL buffer), it will be followed by an invitation to a diagnostic colonoscopy. If any colorectal polyps are detected, they are removed, or a biopsy is taken in case of suspected cancer before referral to further treatment [15].

The Danish CRC Screening Program is managed by the five Danish regions using the joint Invitation and Administration Module (IAM). Since the beginning of the screening program in March 2014, all Danish citizens aged 50–74 have been invited to participate in the program [14]. In this register-based cross-sectional study, we included all first-round screening participants with a positive FIT who were subsequently offered a colonoscopy. The colonoscopies were performed in the period from March 2014 until February 2019.

### 2.2. Data Sources

Data for this study were obtained from several Danish registers on health and population through the Danish Colorectal Cancer Screening Database (DCCSD) and Statistics Denmark. The DCCSD provided data from the Danish CRC Screening Program, which included data from the IAM, the Danish National Patient Register (DNPR) and the National Pathology Register [16]. Statistics Denmark is the governmental institution responsible for collecting data about the Danish society, and for this study they provided data from the DNPR, the Danish National Prescription Registry, the Population’s Education Register, the Income Statistics Register, and the Population Statistics Register. The DNPR holds data and diagnoses of all patient hospital contacts, outpatient as well as inpatient treatments [17]. The Danish National Prescription Registry provides information of redeemed prescription medicine [18]. Information of the highest education achieved by the screening participants was retrieved from the Population’s Education Register [19], and the Income Statistics Register provided data of the income of the screening participants [20]. Information about civil status was obtained through the Population Statistics Register. All data were linked through the unique personal identification number given to all Danish citizens by the Civil Registration System.

### 2.3. Study Variables

Our outcome was the result of the index colonoscopy designated as either complete or incomplete. We divided the ICs into IC due to inadequate bowel preparation and IC due to other causes, including pain, stenosis and other causes, as these were the definitions from the DCCSD. “Other cause” was registered in the DCCSD as a non-explicit indication when incomplete investigation was not caused by either pain, stenosis or inadequate bowel preparation. If no cause was registered, the individual was excluded. The main exposure was SES, which was measured by level of income and education, respectively. The measure used for income was the person equated income that considered the number of children and adults living in the household of each participant. The income was an average of 5 years’ income prior to the screening colonoscopy and was adjusted for inflation. Finally, income was divided into quartiles with the lowest income in the first quartile and the highest income in the fourth quartile. Quartiles were defined within the sample population and therefore the quartiles were of equal size in the study. Education was measured as the highest achieved education up to the screening colonoscopy and stratified into the following categories: basic school, high school/vocational education and higher education.

Covariates in this study included comorbidity, peristalsis-affecting medicine, civil status, age and gender. The updated Charlson comorbidity index by Quan et al. was used for measuring comorbidity [21]. Hospital diagnoses 10 years prior to the screening index colonoscopy were included in the rating and subsequently divided into subgroup ratings of 0, 1, 2 and ≥3, where 0 is no comorbidity and ≥3 is severe comorbidity. Redeemed prescriptions for peristalsis-affecting medicine within the three months prior to the screening colonoscopies were included as a binary covariate. Data on civil status were categorized as either living alone or living with a partner. The information was retrieved for the year in which the colonoscopy was performed. Both gender and age were retrieved from the DCCSD. The age of the participants was the age at the day of the index colonoscopy. Age was divided in groups of <55, 55 to 59, 60 to 64, 65 to 69 and ≥70 years.

### 2.4. Statistical Analysis

Descriptive analyses were performed to determine characteristics of the participants included in the study for each of the two outcomes. Multivariate logistic regression models were performed in a simple model and a full model for each outcome. The simple models were assessing the association between the main exposures, level of income and level of education and each outcome. In addition, the full models include the covariates adjusting for any confounding effect for each outcome. The results are presented as odds ratios (ORs). Significance level was set at 5% and 95% confidence intervals were calculated.

Data management and statistical analyses were performed in Stata version 16.0 (StataCorp LLC, College Station, TX, USA), SAS software version 9.4 (SAS Institute Inc., SAS 9.4., Cary, NC, USA) and R statistical software package version 3.6.1 (R Core Team, Vienna, Austria).

### 2.5. Ethics

All data in this study were stored at Statistics Denmark on secure logged servers and were pseudo-anonymized before the authors were granted access. Therefore, computer code and raw data are not available. The study was approved by the Danish Data Protection Agency through the Region of Southern Denmark (journal 19/32137). Due to the register-based nature of the study, no ethical approvals were necessary.

## 3. Results

**Figure 1:**
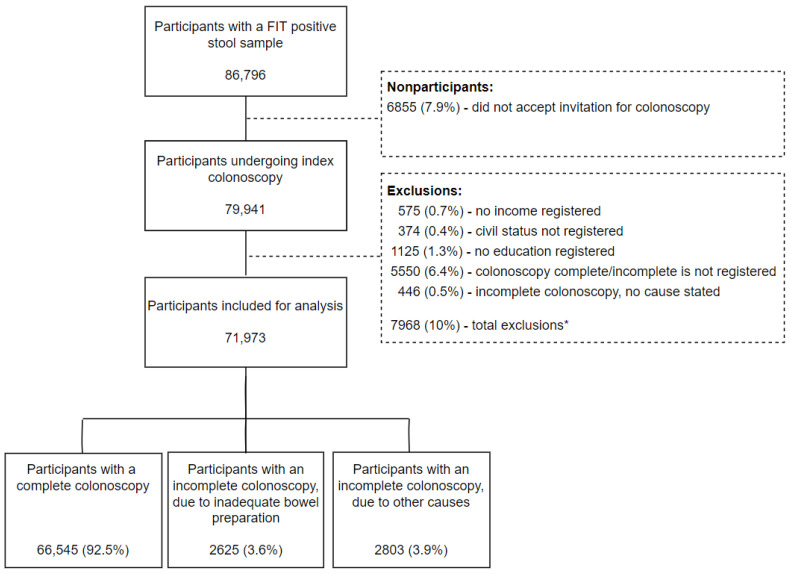
Flowchart of 86,796 first-round participants in the Danish Colorectal Cancer Screening Program with a positive FIT, of which 71,973 were eligible for further analysis. * Participants may be excluded in several variables.

Among the first-round screening participants, 86,796 persons had a positive FIT and were invited to undergo a colonoscopy. A total of 6855 (7.9%) participants did not accept the invitation and dropped out of the study. We excluded 5550 (6.4%) participants where no registration had been made on outcome of the colonoscopy, and 446 (0.5%) participants with no cause stated for the incomplete colonoscopy. A further 575 (0.7%) participants had no income registered, and 1125 (1.3%) had no education registered, which led to exclusion. Finally, civil status was missing for 374 (0.4%) of the screening participants, and they were excluded from the study as well. Thus, 71,973 first-round screening participants were eligible for further analysis. Among these participants, 66,545 (92.5%) had a complete colonoscopy, 2625 (3.6%) participants had an IC due to inadequate bowel preparation, and 2803 (3.9%) participants had an IC due to other causes (Figure 1).

The highest frequency of IC due to inadequate bowel preparation was demonstrated among participants in the lowest income quartile and with the lowest educational level. Similarly, participants with a CCI-score larger than three and participants living alone accounted for a larger proportion of participants with IC along with participants who had redeemed peristalsis-affecting medicine. No significant differences were observed for gender and age (Table 1). In terms of IC due to other reasons, female participants and participants ≥ 70 years had the highest share of ICs. For the remaining categories, a similar pattern was seen as for the IC due to inadequate bowel preparation (Table 2).

**Figure 2:**
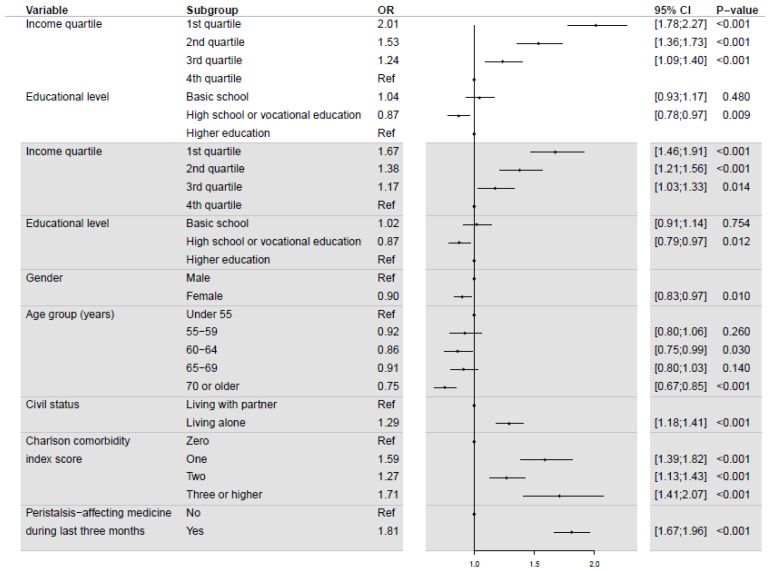
Odds ratios of incomplete colonoscopy due to inadequate bowel preparation from simple (white) and full (grey) logistic regression models.

The simple model for IC due to inadequate bowel preparation showed an OR of 2.01 (95% CI: 1.78; 2.27) for participants with income in the 1st quartile using income in the 4th quartile as reference (Figure 2–white area). For income in the 2nd and 3rd quartiles, the ORs were 1.53 (95% CI: 1.36; 1.73) and 1.24 (95% CI: 1.09; 1.40), respectively. In terms of educational level, the OR for persons with high school/vocational education was 0.87 (95% CI: 0.78; 0.97) compared to participants with higher education. No significant difference was observed for participants with basic school education compared to participants with higher education. In the full model, adjusting for covariates (Figure 2–grey area), the ORs for income and education were similar to the simple model. Participants with income in the 1st quartile had an OR of 1.67 (95% CI 1.46; 1.91) of having an IC due to inadequate bowel preparation compared to participants with income in the 4th quartile. For participants with income in the 2nd and 3rd quartiles the ORs were 1.38 (95% CI: 1.21; 1.56) and 1.17 (95% CI: 1.03; 1.33), respectively. High school/vocational education had an OR of 0.87 (95% CI: 0.79; 0.97) compared to higher education, whereas basic school education was not associated with a significantly higher odds of having an IC due to inadequate bowel preparation.

**Figure 3:**
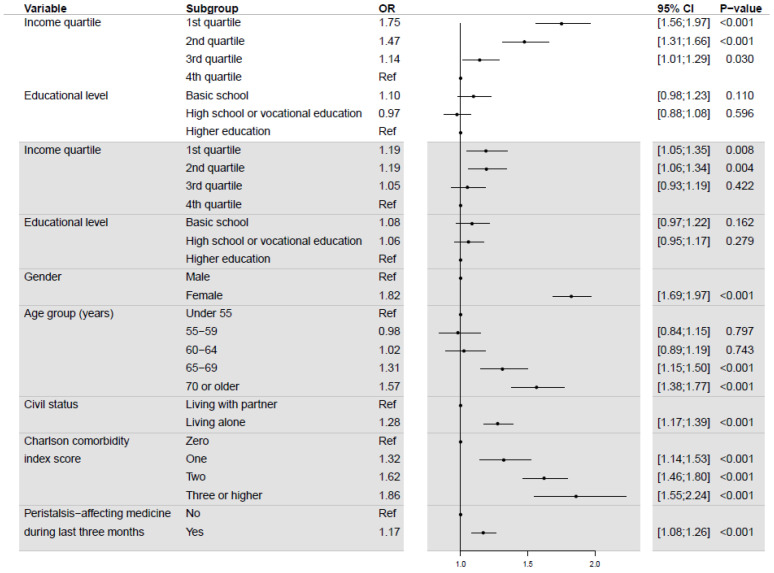
Odds ratios of incomplete colonoscopy due to other reasons from simple (white) and full (grey) logistic regression models.

For the outcome IC due to other reasons, the simple model showed that persons with income in the 1st quartile had an OR of 1.75 (95% CI: 1.56; 1.97) compared to persons with income in the 4th quartile, and for persons with income in the 2nd and 3rd quartiles ORs were 1.47 (95% CI: 1.31; 1.66) and 1.14 (95% CI: 1.01; 1.29), respectively (Figure 3–white area). Educational level was not associated with significantly different odds of having an IC due to other reasons. In terms of the full model (Figure 3–grey area), the trend of the ORs for income changed compared to the simple model. ORs for income in the lowest two quartiles are both 1.19 (95% CI: 1.05; 1.35) and (95% CI: 1.06; 1.34) compared to the 4th quartile, and for participants with income in the 3rd quartile no significance was shown. The level of education was not associated with significantly different odds of having an IC due to other reasons in the full model.

## 4. Discussion

Lower income quartiles were associated with increased odds of IC due to poor bowel preparation for first round participants in the Danish CRC Screening Program. For the 1st income quartile, the OR was 2.01 in the simple model and 1.67 in the fully adjusted model, when compared to the 4th income quartile. The two lowest income quartiles had increased odds of IC due to other causes. The associations between education and IC due to inadequate bowel preparation and due to other causes were inconsistent. This indicates that education is not as strongly associated with IC as income. Both the WHO and Friis et al. state that lower level of education is associated with poor health literacy [22,23], and other studies have shown that poor health literacy predicts inadequate bowel preparation [24,25]. This is, however, not in accordance with the findings of this study. It may still be true that health literacy is associated with poor bowel preparation, but this trend is not transferred to educational level in our study.

SES is known to be associated with comorbidity [10]. Having an inflammatory bowel disease or taking certain psychoactive drugs has shown to affect bowel cleanliness [26,27]. This study considered comorbidity in a more general way by using the CCI, but diseases like inflammatory bowel disease and mental diseases are not included in the CCI [21]. We have taken into account the redeemed prescriptions of peristalsis affecting medicine of the participants, which is relevant for the process of bowel preparation prior to colonoscopy. However, there is minimal risk of misclassification as the intake of the peristalsis affecting medicine is not ensured just because the participants redeemed their prescription. Further studies detailing the link between comorbidity and IC are planned.

Social inequality has been demonstrated despite the fact that the Danish CRC Screening Program invites all citizens aged 50–74 years and the program is free of charge [14]. Studies have shown that low SES is associated with lower participation rate compared to higher SES [11,28], and participants with the highest level of education and the highest level of income, respectively, submit the lowest proportion of ineligible FIT samples [13]. Additionally, socioeconomic inequality is evident in adherence to follow-up diagnostic colonoscopies [12]. This is in line with our findings, when SES is measured by income, and consequently, there is an increased and accumulated inequality in the Danish CRC Screening Program, where participants with high SES do best through each stage of the screening program.

Additionally, we do not know whether the poor bowel preparation in the deprived might be due to poor compliance with the bowel preparation procedure, or because the bowel preparation procedure did not have a sufficient effect. Future studies may clarify this and thus help reduce the number of ICs.

By identifying individuals at risk of an IC before the colonoscopy, it may be possible to take countermeasures and save these persons for an inconvenient extra bowel preparation and examination while preventing unnecessary extra costs and use of resources. An additional effect might be a reduction in the social inequality in the Danish CRC Screening Program.

Since the beginning of the Danish CRC Screening Program, the participation rate has been 59–64% [15,29], and because it is shown that lower SES predicts lower participation [11], this may cause a non-response bias. The result of this study is therefore limited to the participating population and not necessarily transferable to the complete population. Thus, the ORs are more likely to be underestimated than the opposite, as the lower SES subgroups are less well represented. As this was a register-based study, not all relevant variables are available, such as BMI. For this to bias our results, the BMI would need to differ between educational and income groups, which is plausible.

A strength of this study was the large sample size based on participants in the organized Danish CRC Screening Program. Since all Danish citizens in the target group were invited, the risk of selection bias is minimal. However, if missing data are not missing at random, there is a risk of selection bias. An additional strength was that data for this study were retrieved from validated Danish national registers with a single central identifier that links all registers.

## 5. Conclusions

Low income was associated with increased odds of IC caused by poor bowel preparation and other causes defined by pain, stenosis and other causes. Low educational level was not associated with increased odds of IC, except for individuals with a high school/vocational education, who were at decreased odds of IC due to poor bowel preparation.

## Figures and Tables

**Table 1:** Characteristics of the study population stratified by complete colonoscopy or incomplete colonoscopy due to inadequate bowel preparation.

Inadequate Bowel Preparation					
Variables	Level	Complete (%) * *n* = 66,545	Incomplete (%) * *n* = 2625	Total (%) ** *n* = 69,170	*p*-Value
Income quartile	1st quartile	15,947 (94.7)	892 (5.3)	16,839 (24.3)	
	2nd quartile	16,489 (96.0)	691 (4.0)	17,180 (24.8)	
	3rd quartile	16,938 (96.7)	570 (3.3)	17,508 (25.3)	
	4th quartile	17,171 (97.3)	472 (2.7)	17,643 (25.5)	<0.001
Educational level	Basic school	18,424 (95.4)	890 (4.6)	19,314 (27.9)	
	High school or vocational education	31,879 (96.5)	1151 (3.5)	33,030 (47.8)	
	Higher education	16,242 (96.5)	584 (3.5)	16,826 (24.3)	<0.001
Gender	Male	37,806 (96.3)	1460 (3.7)	39,266 (56.8)	
	Female	28,739 (96.1)	1165 (3.9)	29,904 (43.2)	0.2338
Age group (years)	Under 55	11,365 (96.1)	467 (3.9)	11,832 (17.1)	
	55–59	9286 (96.4)	349 (3.6)	9635 (13.9)	
	60–64	11,957 (96.5)	433 (3.5)	12,390 (17.9)	
	65–69	14,508 (96.0)	601 (4.0)	15,109 (21.9)	
	70 or older	19,429 (96.2)	775 (3.8)	20,204 (29.2)	0.1940
Charlson comorbidity	Zero	53,442 (96.6)	1884 (3.4)	55,326 (80.0)	
index score	One	3899 (93.7)	263 (6.3)	4162 (6.0)	
	Two	7512 (95.5)	355 (4.5)	7867 (11.4)	
	Three or higher	1692 (93.2)	123 (6.8)	1815 (2.6)	<0.001
Civil status	Living with partner	48,444 (96.7)	1660 (3.3)	50,104 (72.4)	
	Living alone	18,101 (94.9)	965 (5.1)	19,066 (27.6)	<0.001
Peristalsis-affecting medicine	No	40,414 (97.2)	1143 (2.8)	41,557 (60.1)	
during last three months	Yes	26,131 (94.6)	1482 (5.4)	27,613 (39.9)	<0.001

* Proportion within variable level. ** Proportion within total population.

**Table 2:** Characteristics of the study population stratified by incomplete colonoscopy due to other reasons.

Other Reasons					
Variables	Level	Complete (%) * *n* = 66,545	Incomplete (%) * *n* = 2803	Total (%) ** *n* = 69,348	*p*-Value
Income quartile	1st quartile	15,947 (94.7)	896 (5.3)	16,843 (24.3)	
	2nd quartile	16,489 (95.5)	768 (4.5)	17,257 (24.9)	
	3rd quartile	16,938 (96.6)	605 (3.4)	17,543 (25.3)	
	4th quartile	17,171 (97.0)	534 (3.0)	17,705 (25.5)	<0.001
Educational level	Basic school	18,424 (95.3)	918 (4.7)	19,342 (27.9)	
	High school or vocational education	31,879 (96.1)	1287 (3.9)	33,166 (47.8)	
	Higher education	16,242 (96.4)	598 (3.6)	16,840 (24.3)	<0.001
Gender	Male	37,806 (97.0)	1152 (3.0)	38,958 (56.2)	
	Female	28,739 (94.6)	1651 (5.4)	30,390 (43.8)	<0.001
Age group (years)	Under 55	11,365 (96.9)	368 (3.1)	11,733 (16.9)	
	55–59	9286 (97.0)	292 (3.0)	9578 (13.8)	
	60–64	11,957 (96.8)	394 (3.2)	12,351 (17.8)	
	65–69	14,508 (95.8)	643 (4.2)	15,151 (21.9)	
	70 or older	19,429 (94.6)	1106 (5.4)	20,535 (29.6)	<0.001
Charlson comorbidity	Zero	53,442 (96.5)	1962 (3.5)	55,404 (79.9)	
index score	One	3899 (94.6)	224 (5.4)	4123 (5.9)	
	Two	7512 (94.0)	482 (6.0)	7994 (11.5)	
	Three or higher	1692 (92.6)	135 (7.4)	1827 (2.6)	<0.001
Civil status	Living with partner	48,444 (96.4)	1798 (3.6)	50,242 (72.4)	
	Living alone	18,101 (94.7)	1005 (5.3)	19,106 (27.6)	<0.001
Peristalsis-affecting medicine	No	40,414 (96.5)	1464 (3.5)	41,878 (60.4)	
during last three months	Yes	26,131 (95.1)	1339 (4.9)	27,470 (39.6)	<0.001

* Proportion within variable level. ** Proportion within total population.

## Data Availability

All data in this study were stored at Statistics Denmark on secure logged servers and was pseudo-anonymized before the authors were granted access, therefore computer code and data are not available.
